# Categorical Data Analysis for High-Dimensional Sparse Gene Expression Data

**DOI:** 10.3390/biotech12030052

**Published:** 2023-07-27

**Authors:** Niloufar Dousti Mousavi, Hani Aldirawi, Jie Yang

**Affiliations:** 1 Department of Mathematics, Statistics, and Computer Science, University of Illinois at Chicago, Chicago, IL 60607, USA; sdoust2@uic.edu; 2 Department of Mathematics, California State University—San Bernardino, San Bernardino, CA 92407, USA; hani.aldirawi@csusb.edu

**Keywords:** multinomial logistic model, zero-inflated model, hurdle model, model selection, variable selection, order selection, cross-validation

## Abstract

Categorical data analysis becomes challenging when high-dimensional sparse covariates are involved, which is often the case for omics data. We introduce a statistical procedure based on multinomial logistic regression analysis for such scenarios, including variable screening, model selection, order selection for response categories, and variable selection. We perform our procedure on high-dimensional gene expression data with 801 patients, 2426 genes, and five types of cancerous tumors. As a result, we recommend three finalized models: one with 74 genes achieves extremely low cross-entropy loss and zero predictive error rate based on a five-fold cross-validation; and two other models with 31 and 4 genes, respectively, are recommended for prognostic multi-gene signatures.

## 1. Introduction

High-dimensional data is a dataset in which the number of covariates (*d*) is much larger than the number of observations (*n*), often written as d≫n. It is raised in many scientific fields, especially in biological sciences [1,2,3], where the datasets are difficult to analyze using classical statistical tools such as linear, nonlinear, or generalized linear regression models. When the covariates have a high percentage of zeros, it is necessary to use zero-inflated models or hurdle models to address this sparsity [4,5,6]. The analysis becomes more challenging due to the skewness of the distributions [7].

High-dimensional sparse data is wide-ranging. It arises in many different disciplines such as genomics [8], mobile app usage logs [2], and energy technologies [9]. Omics data such as microbiome [10] and gene expression data [11] are typically high-dimensional and sparse.

As a motivating example, the RNA-seq gene expression data discussed in [1] consists of n=801 tissue samples with 20,531 genes. Among them, 2426 genes carry a good proportion of zeros, from 5% to 50%. The goal of their analysis was to predict the response category which belongs to one of the five different types of cancerous tumors, namely BRCA, COAD, KIRC, LUAD, and PRAD, based on the 2426 sparse genes. A variable selection criterion was proposed by [1] to rank the 2426 sparse genes for predicting the type of tumors, and the top 50 genes were recommended with a 1-nearest neighbor classifier, which is highly robust for high-dimensional classifications [12].

Unlike statistical methods, the 1-nearest neighbor classifier, as well as many other machine learning techniques, is a deterministic classification approach, which assigns a single predictive label to each given subject or individual. Statistical methods, however, typically produce a distribution answer, called a stochastic classification [13], which assigns a probability to each possible response category or label. In general, a stochastic classification result provides more information than a deterministic one, especially for mixed cases or cases close to a boundary between classes.

In this paper, we consider statistical models and stochastic classifications for high-dimensional sparse data with categorical responses. If the response is binary, generalized linear models [14,15] have been widely used in practice. When the response has three or more categories, such as the five tumor types in the motivating example of gene expression data, we consider the multinomial logistic models [16,17,18], which include four commonly used logit models, baseline-category, cumulative, adjacent-categories, and continuation-ratio (please see [18] for a good review and the references therein).

## 2. Material and Method

In this section, we introduce the multinomial logistic models and order selection for categorical responses, a variable screening technique for sparse covariates, and variable selection techniques.

### 2.1. Multinomial Logisitic Models

In this paper, we suppose that the original data takes the form of $\left\{\left(x_{i},y_{i}\right),i=1,\ldots ,n\right\}$ with covariates $x_{i}={\left(x_{i1},\ldots ,x_{id}\right)}^{T}\in R^{d}$, d≥1 and categorical response $y_{i}\in \left\{1,\ldots ,J\right\}$, J≥2. We further assume that there are only *m* distinct $x_{i}$ vectors, and denote them as $x_{1},\ldots ,x_{m}$, m≤n for simplicity. For applications with discrete covariates only, it is often the case that m≪n. Following the notations in [18], we consider the data in its summarized form $\left\{\left(x_{i},Y_{i}\right),i=1,\ldots ,m\right\}$, where $Y_{i}={\left(Y_{i1},\ldots ,Y_{iJ}\right)}^{T}$, with $Y_{ij}=\#\{k|x_{k}=x_{i}$$andy_{k}=j\}$, the number of observations with covariates $x_{i}$ and response category *j*. We denote $n_{i}=\sum_{j=1}^{J}Y_{ij}$, the number of observations with covariate $x_{i}$, i=1,…,m.

Following [18,19], a multinomial logistic model assumes that *(i)* $Y_{i}$ follows a multinomial distribution with number $n_{i}$ and category probabilities $\pi_{i1},\ldots ,\pi_{iJ}$ independently for different *i*, where $\sum_{j=1}^{J}\pi_{ij}=1$; *(ii)* the category probabilities $\pi_{ij}$ are linked to functions of covariates in one of the four following ways:

(1) $$\begin{array}{ccc} \log\left(\frac{\pi_{ij}}{\pi_{iJ}}\right) & = & h_{j}^{T}\left(x_{i}\right)\beta_{j}+h_{c}^{T}\left(x_{i}\right)\zeta ,\;\mathrm{baseline}-\mathrm{category} \end{array}$$

(2) $$\begin{array}{ccc} \log\left(\frac{\pi_{i1}+\cdots +\pi_{ij}}{\pi_{i,j+1}+\cdots +\pi_{iJ}}\right) & = & h_{j}^{T}\left(x_{i}\right)\beta_{j}+h_{c}^{T}\left(x_{i}\right)\zeta ,\;\mathrm{cumulative} \end{array}$$

(3) $$\begin{array}{ccc} \log\left(\frac{\pi_{ij}}{\pi_{i,j+1}}\right) & = & h_{j}^{T}\left(x_{i}\right)\beta_{j}+h_{c}^{T}\left(x_{i}\right)\zeta ,\;\mathrm{adjacent}-\mathrm{categories} \end{array}$$

(4) $$\begin{array}{ccc} \log\left(\frac{\pi_{ij}}{\pi_{i,j+1}+\cdots +\pi_{iJ}}\right) & = & h_{j}^{T}\left(x_{i}\right)\beta_{j}+h_{c}^{T}\left(x_{i}\right)\zeta ,\;\mathrm{continuation}-\mathrm{ratio} \end{array}$$

Here $h_{j}^{T}\left(\cdot \right)=\left(h_{j1}\left(\cdot \right),\ldots ,h_{jp_{j}}\left(\cdot \right)\right)$ and $h_{c}^{T}\left(\cdot \right)=\left(h_{1}\left(\cdot \right),\ldots ,h_{p_{c}}\left(\cdot \right)\right)$ are known predictor functions; $\beta_{j}={\left(\beta_{j1},\ldots ,\beta_{jp_{j}}\right)}^{T}$ and $\zeta ={\left(\zeta_{1},\ldots ,\zeta_{p_{c}}\right)}^{T}$ are unknown regression parameters, i=1,…,m, j=1,…,J−1. It should be noted that when J=2, all four logit models, namely baseline-category (1), cumulative (2), adjacent-categories (3), and continuation-ratio (4), are equivalent to the logistic regression model for binary responses.

In this paper, we consider two special classes of multinomial logistic models, *proportional odds* (po) models assuming $h_{j}^{T}\left(x_{i}\right)\equiv 1$, i.e., the same parameters for different categories except the intercepts, and *nonproportional odds* (npo) models assuming $h_{c}^{T}\left(x_{i}\right)\equiv 0$, i.e., different parameters across categories. The four link models (1), (2), (3), and (4), combined with either the po or npo assumption, lead to eight different models. For example, one model is called an adjacent-categories po model if it is an adjacent-categories logit model (3) with the po assumption. In practice, people often adopt $h_{c}\left(x_{i}\right)=x_{i}$ for po models and $h_{j}\left(x_{i}\right)={\left(1,x_{i}^{T}\right)}^{T}$ for npo models, also known as main-effects models. More general models and examples can be found in [18].

### 2.2. The Most Appropriate Order for Categorical Responses

In the statistical literature, the baseline-category logit model (1) is also known as a multiclass logistic regression model (see, for example, [20]). It is commonly used for nominal responses, i.e., the response categories do not have a natural ordering [21]. In model (1), the *J*th category is regarded as the baseline category. The choice of the baseline category is known to be irrelevant for prediction purposes for npo models. Nevertheless, as pointed out by [19], the baseline category needs to be carefully chosen for po models, since it may improve the prediction accuracy significantly. We adopt the Akaike information criterion (AIC, [20,22]) for choosing the most appropriate baseline category for baseline-category po models.

Models (2), (3), and (4) are typically used for ordinal or hierarchical responses, which assume either a natural ordering or a hierarchical structure among the response categories. Surprisingly, according to [19], even for responses whose categories do not have a natural or known order, one may still use AIC or Bayesian information criterion (BIC, see also [20]) to choose the most appropriate order, called a *working* order, for the response categories. Then models (2), (3), and (4) can also be used for nominal responses with the working order, which may significantly improve the prediction accuracy.

In this paper, we use AIC to choose the most appropriate (working) order for the five tumor types in the motivating example so that all the four logit models can be applied.

### 2.3. Sparse Variable Screening Using the AZIAD Package

To fit a multinomial logistic model and obtain the parameter estimates with the corresponding confidence intervals, we need the number *m* of distinct $x_{i}$ vectors to be large enough to keep the corresponding Fisher information matrix positive definite. According to [18], the smallest possible *m* is $p_{c}+1$ for po models or $\max\{p_{1},\ldots ,p_{J-1}\}$ for npo models. If we consider main-effects models only, then m≥d+1 is required for both po and npo models. In other words, we need to reduce the number *d* of covariates below m−1 before fitting a multinomial logistic model.

For the motivating example, following [1], we focus on the 2426 sparse genes. In this case, n=m=801, since the gene expression levels are continuous. To reduce the number of genes below 800, we first calculate a rank of all these genes based on the AIC differences of the significance test proposed by [1], which involves model selections from a list of candidate distributions based on the *p*-values of KS-tests using the R package AZIAD [23]. More specifically, for each gene, we consider two probabilistic models. In Model I, we assume that all 801 gene expression levels follow the same probability distribution chosen from a candidate set; while in Model II, we divide the 801 numbers into five groups according to their response labels and assume that each group of numbers follows a distinct distribution. We fit both models and denote the corresponding AIC values as AIC(I) and AIC(II), respectively. According to [1], a bigger difference between AIC(I) and AIC(II) indicates that the gene is more informative for predicting the response labels.

Since the gene expression levels are continuous and non-negative, we consider a candidate set of 12 probability distributions, including normal (or Gaussian), zero-inflated normal, normal hurdle, half-normal, zero-inflated half-normal, half-normal hurdle, log-normal, zero-inflated log-normal, log-normal hurdle, exponential, zero-inflated exponential, and exponential hurdle distributions (please see [7] for a complete list of distributions available in AZIAD). As a result, we obtain a rank of the 2426 sparse genes from high to low, corresponding to their AIC differences from high to low. We then use a five-fold cross-validation to select the best number of genes for fitting multinomial logistic models (see Section 2.4).

### 2.4. Best Number of Covariates for Categorical Regression Analysis

In [1], a five-fold cross-validation with a 1-nearest neighbor classifier was used for choosing the best number of genes. In other words, a predictive error count or error rate was used as the criterion for variable selection.

When a stochastic classification is available, the cross-entropy loss, which measures the difference between the predictive probabilities based on the fitted model and the observed class labels, is more commonly used as a criterion and is more sensitive for variable selection or model selection [20].

In this paper, we choose the number of ranked covariates that minimizes the cross-entropy loss based on a five-fold cross-validation. More specifically, given a specified regression model and the top *t* covariates under consideration, (1) we randomly divide the original *n* observations (not the summarized data) into five data blocks of roughly the same size, then for each i=1,…,n, the *i*th observation belongs to one and only one of the five data blocks, denoted as the k(i)th block; (2) for each k=1,…,5, we fit the regression model with the top *t* covariates based on the four data blocks other than the *k*th one, denoted as the *k*th fitted model; (3) for each i=1,…,n, we predict $\pi_{ij}$, j=1,…,J based on the k(i)th fitted model and denote the predictive probability as ${\hat{\pi }}_{ij}^{k(i)}$; and (4) the (average) cross-entropy loss for the specified model and the given number *t* is defined as

(5) $$\mathrm{CE}\left(t\right)=-\frac{1}{n}\sum_{i=1}^{n}\log\left({\hat{\pi }}_{i,y_{i}}^{k(i)}\right)$$

For the specified model, we choose the *t* that minimizes CE(t).

### 2.5. Backward Variable Selection Based on AIC

Backward variable selection aims to identify a good (if not the best) subset of variables by iteratively removing the least-significant predictor from a statistical model. Typically, AIC is used as the criterion for eliminating a variable (see, for example, Section 3.3 in [20]). In our case, (1) we fit a specified multinomial logistic model with the top *t* covariates obtained in Section 2.4 and record its AIC value as AIC(t); (2) we initialize r=t to be the number of covariates in the model and perform the following steps while r≥1; (3) for each s=1,…,r, we fit the regression model with the *s*th covariates removed and denote the corresponding AIC value as AIC(r−1,s); (4) we let $\mathrm{AIC}\left(r-1\right)=\min_{s}\left\{\mathrm{AIC}\left(r-1,s\right)\right\}$, the smallest AIC value with r−1 covariates; and (5) if AIC(r−1)<AIC(r) and r≥1, we remove the covariate that attains AIC(r−1) and go back to step (3) with *r* replaced with r−1, otherwise we stop and report the model with the current set of *r* covaraites.

It should be noted that there are other strategies for selecting a subset of covariates, such as forward selection or forward and backward selection. We refer [20] to the readers for more options.

## 3. RNA-Seq Gene Expression Data

The illustrative example that we use in this paper is a high-dimensional RNA-seq gene expression dataset. The original dataset consists of n=801 tissue samples and 20,531 genes with five response categories (and frequencies), namely BRCA (300), COAD (78), KIRC (146), LUAD (141), and PRAD (136) [1,24]. The data can be downloaded from the UCI Machine Learning Repository (https://archive.ics.uci.edu/ml/datasets/gene+expression+cancer+RNA-Seq#, accessed on 4 November 2022).

Following [1], we consider only the 2426 genes whose expression levels have a proportion of zeros between 5% and 50%, which is still high-dimensional. Since the gene expression levels are continuous, all 801 $x_{i}$ vectors are distinct and thus n=m=801 in our notations (see also Section 2.1). Applying the significance test described in [1] to each of the genes (see also Section 2.3), we obtain a rank of the 2426 genes from high to low in terms of relevance to the five response categories. Based on a five-fold cross-validation with the 1-nearest neighbor classifier, the top 50 genes were recommended by [1] for predicting the response categories. The 1-nearest neighbor classifier based on the selected 50 genes achieves zero predictive error rate based on the five-fold cross-validation.

In this paper, we use this data to illustrate how to use the proposed model selection procedure to build up the most appropriate regression model for analyzing a high-dimensional sparse data with categorical responses.

## 4. Data Analysis and Results

Through this comprehensive analysis on the RNA-seq gene expression data, we aim to identify the most appropriate regression model with selected genes that would provide valuable insights into the relationship between the gene expression levels and the type of cancers.

### 4.1. Model Selection and Variable Selection for Sparse Genes

We first use the R function vglm in package VGAM [25,26] to fit eight candidate multinomial logistic main-effects models (see Section 2.1) given different numbers of selected genes including the baseline-category po model (nompo), the baseline-category npo model (nomnpo, also known as the multiclass logistic regression model), the cumulative po model (cumpo), the cumulative npo model (cumnpo), the continuation-ratio po model (crpo), the continuation-ratio npo model (crnpo), the adjacent-categories po model (acpo), and the adjacent-categories npo model (acnpo). The R codes for the top 80 ranked sparse genes are provided below, where yj stands for the vector ${\left(Y_{1j},\ldots ,Y_{nj}\right)}^{T}$, j=1,…,5.

library(VGAM)

data = varselect80

fit.nompo <- vglm(cbind(y1,y2,y3,y4,y5) ~ .,

familyy = multinomial(parallel=T), data = data)

fit.nomnpo <- vglm(cbind(y1,y2,y3,y4,y5) ~ .,

familyy = multinomial, data = data)

fit.cumpo <- vglm(cbind(y1,y2,y3,y4,y5) ~ .,

familyy = cumulative(parallel=T), data = data)

fit.cumnpo <- vglm(cbind(y1,y2,y3,y4,y5) ~ .,

familyy = cumulative, data = data)

fit.crpo <- vglm(cbind(y1,y2,y3,y4,y5) ~ .,

familyy = sratio(parallel=T), data = data)

fit.crnpo <- vglm(cbind(y1,y2,y3,y4,y5) ~ .,

familyy = sratio, data = data)

fit.acpo <- vglm(cbind(y1,y2,y3,y4,y5) ~ .,

familyy = acat(parallel=T), data = data)

fit.acnpo <- vglm(cbind(y1,y2,y3,y4,y5) ~ .,

familyy = acat(reverse=T), data = data)

The best models out of the eight candidates, along with their cross-entropy losses and predictive error counts/rates based on a five-fold cross-validation, are listed in Table 1. Here, the cross-entropy losses are calculated as described in (5) with the predictive probabilities $\left({\hat{\pi }}_{i1}^{k(i)},\ldots ,{\hat{\pi }}_{i5}^{k(i)}\right)$ obtained from the k(i)th fitted model, while the predictive error counts are calculated as $\#\{i|y_{i}\neq {\hat{y}}_{i}\}$, where ${\hat{y}}_{i}={\mathrm{argmax}}_{j}{\hat{\pi }}_{ij}^{k(i)}$, i.e., the *j* with the largest predictive probability. For this dataset, three models, the adjacent-categories npo model (or adjacent-cate. npo model in short), the baseline-category npo model, and the adjacent-categories po model (or adjacent-cate. po model in short) frequently appear among the top. According to Table 1, we choose the top t=80 genes as the start. Compared with t=50, t=80 is a more conservative choice since we will perform backward variable selections as the next step anyway.

### 4.2. Order Selection for Response Categories

At this stage of the proposed analysis, we keep three candidate models under consideration: the adjacent-categories po model, the adjacent-categories npo model, and the baseline-category npo model. According to [19], the order of the response categories matters only for the adjacent-categories po model (see also Section 2.2).

To find the most appropriate order of response categories for the adjacent-categories po model in this case, we explore all the 5!=120 different orders of y1, y2, y3, y4 and y5. For each order, we fit the adjacent-categories po model with the top 80 genes and record the corresponding AIC value. The best order, which achieves the smallest AIC value, is (y2, y3, y4, y1, y5), which corresponds to (COAD, KIRC, LUAD, BRCA, PRAD) with AIC value 175.81, while the AIC value with the original order is 309.52. The improvement is significant [27]. It should be noted that the reversed order of the best one achieves the same AIC as well [19].

### 4.3. Backward Variable Selected Models

In this section, we apply the backward variable selection procedure (see Section 2.5) to the three candidate models, starting with the top t=80 genes.

We start with the adjacent-categories po model with order (y2, y3, y4, y1, y5) and AIC=176. After backward variable selection, we end with 31 genes and AIC value 90. As for the adjacent-categories npo model, we start with 80 genes and AIC value 648 and end with 74 genes and AIC value 600. We also run the backward variable selection for the baseline-category npo model (i.e., the multiclass logistic regression model). Its 80 genes are reduced to only 4 genes, along with AIC values from 648 to 52. The backward variable selection improves all the three models significantly in terms of AIC values [27].

As a summary, after the backward variable selection, our three candidate models are reduced as (1) an adjacent-categories po model with 31 genes; (2) an adjacent-categories npo model with 74 genes; and (3) a baseline-category npo model with 4 genes. It should be noted that two genes, *gene_7965* and *gene_9176*, appear in all the three final models.

### 4.4. Final Models

We further evaluate the performance of the three final models obtained in Section 4.3, whose cross-entropy losses and predictive error counts/rates based on a five-fold cross-validation are provided in Table 2. In terms of prediction accuracy, the adjacent-categories npo model with 74 genes is clearly the winner since it achieves the lowest cross-entropy loss of $2.2\times 10^{-9}$ and error count of 0. For readers’ reference, we provide the adjacent-categories npo model with 74 genes fitted on all 801 samples in Appendix C.

Nevertheless, if one aims to find a prognostic multi-gene signature (see Section 4.5 for more details) for predicting the risk of the five types of cancers, the remaining two models have their values as well, since they require information from a much smaller number of genes. As a comparison, all the three final models are much more accurate than the best model based on the top 50 genes recommended by [1], which is an adjacent-categories npo model with a five-fold cross-entropy loss of 0.14 and error rate of 0.0087 (see Table 1). The final adjacent-categories po model with 31 genes misclassifies the case *sample_190* with predictive probabilities $\left(1.04\times 10^{-13},\;2.68\times 10^{-10},\;0.576,\;0.424,\;2.95\times 10^{-37}\right)$. Although the observed label of *sample_190* is type 4 (or LUAD), the predictive probability 0.424 is about 0.5, i.e., at the boundary between type 3 and type 4. We can even further reduce the multi-gene signature to a set of four genes, *gene_7965*, *gene_9176*, *gene_12977*, and *gene_15898*. With the final baseline-category npo model, we can still achieve high predictive accuracy (801−2)/801=99.75% based on a five-fold cross-validation.

### 4.5. Prognostic Multi-Gene Signatures

A *prognostic multi-gene signature* is a set of genes whose expression patterns can serve as a prognostic biomarker for the associated phenotype or condition, such as the cancer types in our example [28,29]. In this section, we compare four potential prognostic multi-gene signatures: (1) the 31 genes (see Appendix A) selected for the adjacent-categories po (acpo) model; (2) the 74 genes (see Appendix B) selected for the adjacent-categories npo (acnpo) model; (3) the 4 genes (see Section 4.4) selected for the baseline-category npo (nomnpo) model; and (4) the top 50 genes (top50) recommended by [1].

In Table 3, we list the number of genes shared by the row multi-gene signature and the column one. As mentioned in Section 4.3, two genes, *gene_7965* and *gene_9176*, are shared by acpo, acnpo and nomnpo, while only one gene, *gene_9176*, is shared by all four signatures. It should be noted that the genes selected for acpo and nomnpo are neither subsets of acnpo nor top50. They overlap with each other, but without inclusion.

## 5. Discussion

In this paper, we propose a data analysis procedure for a high-dimensional sparse data with categorical response variables, including covariate screening, model selection, order selection, and variable selection. Different from typical machine learning techniques, the prediction given the covariates is a probability distribution on the collection of response categories. For example, the prediction for *sample_190* in the RNA-seq gene expression data is $\left(1.04\times 10^{-13},\;2.68\times 10^{-10},\;0.576,\;0.424,\;2.95\times 10^{-37}\right)$ based on the adjacent-categories po model with 31 genes. Although the observed label is type 4 (or LUAD), the prediction should be interpreted as high risks in both KIRC (type 3) and LUAD since both predictive probabilities are close to 0.5. It is important in practice, since the doctor may suggest the patient receive early screening examinations on both types of tumors. If, instead, the prediction is a deterministic one, in this case, KIRC, whose predictive probability 0.576 is the highest, then the risk of LUAD is ignored by mistake. In other words, a distribution prediction or stochastic classification is much more informative than a deterministic classification.

As mentioned in Section 4.3, Section 4.4 and Section 4.5, we recommend three predictive models for the RNA-seq gene expression data at different levels of cost and accuracy. For predicting the risks of five types of cancers, Model (3) based on four genes costs the least and achieves the lowest accuracy level. In practice, it may be used for general cancer screening purposes. Model (2), based on 74 genes, has the highest predictive accuracy and may be used for clinical diagnosis. Model (1), with 31 genes, stands in the middle and may be used for cancer screening practice for people with a higher risk due to a family history of cancers. In other words, each recommended model may have its own appropriate applications.

Another interesting result obtained in our analysis is the order (COAD, KIRC, LUAD, BRCA, PRAD) chosen for the adjacent-categories po model. According to the reduction in the AIC value from 309.52 to 175.81, the chosen order works significantly better than the original order for the adjacent-categories po model. It may be worth exploring whether the selected order has any implication since it is supported by the data.

It should be noted that, for illustration purposes only, the original gene expression levels $x_{ij}$ variables are used as predictors in Section 4, known as main-effects models. In practice, however, transformations of covariates such as $x_{ij}^{2}$ and $\log(x_{ij}+1)$, or interactions of covariates such as $x_{ij}x_{ik}$ may be considered as potential predictors as well. In that case, the selected final models are expected to be different.

Besides the RNA-seq gene expression data or other omics data in medical sciences, the proposed statistical approach can also be used for high-dimensional sparse data arising in many other scientific disciplines, including survey and demographic data in social sciences, bag-of-words representations of text in natural language processing and information retrieval, mobile app usage logs in application usage analytics, recommender systems in artificial intelligence (AI), etc. [2].

## Figures and Tables

**Table 1:** Best models with top *t* sparse genes based on five-fold cross-validation.

Top *t* Genes	Best Models	Cross-Entropy Loss	Error Count/Rate
25	Baseline-category npo	1.50	40/801 = 0.049
30	Adjacent-cate. npo	0.59	11/801 = 0.013
	Baseline-category npo	0.59	11/801 = 0.013
50	Adjacent-cate. po	1.88	58/801 = 0.072
	Adjacent-cate. npo	0.14	7/801= 0.0087
	Baseline-category npo	0.33	7/801 = 0.0087
60	Adjacent-cate. po	2.01	60/801 = 0.075
	Adjacent-cate. npo	0.15	6/801 = 0.0075
	Baseline-category npo	0.15	6/801 = 0.0075
70	Adjacent-cate. po	1.95	60/801 = 0.075
	Adjacent-cate. npo	0.21	5/801 = 0.0062
	Baseline-category npo	0.21	5/801 = 0.0062
80	Adjacent-cate. po	2.01	70/801 = 0.087
	Adjacent-cate. npo	0.14	4/801 = 0.0049
	Baseline-category npo	0.22	4/801 = 0.0049
100	Adjacent-cate. po	1.79	60/801 = 0.075
150	Adjacent-cate. po	2.41	69/801 = 0.086

**Table 2:** Performance of final models based on five-fold cross-validation.

Number of Genes	Type of Model	Cross-Entropy Loss	Error Count/Rate
31	Adjacent-cate. po	0.063	1/801 = 0.0012
74	Adjacent-cate. npo	$2.2\times 10^{-9}$	0
4	Baseline-category npo	0.031	2/801 = 0.0025

**Table 3:** Counts of genes shared by different multi-gene signatures.

	acpo	acnpo	nomnpo	top50
acpo	31	29	2	20
acnpo	29	74	4	45
nomnpo	2	4	4	2
top50	20	45	2	50

Note: acpo: adjacent-categories po’s 31 genes; acnpo: adjacent-categories npo’s 74 genes; nomnpo: baseline-category npo’s 4 genes; top50: top 50 genes in [1].

## Data Availability

The gene expression data are publicly available from the UCI Machine Learning Repository (https://archive.ics.uci.edu/ml/datasets/gene+expression+cancer+RNA-Seq#, accessed on 4 November 2022) (see also Section 3).

## Appendix A. List of 31 Selected Genes for the Adjacent-Categories Po Model (See Section 4.3 and Section 4.5)

*gene_1054*, *gene_2288*, *gene_2639*, *gene_3439*, *gene_3598*, *gene_3737*, *gene_4467*,

*gene_5050*, *gene_6838*, *gene_7235*, *gene_7964*, *gene_7965*, *gene_8003*, *gene_8349*,

*gene_9175*, *gene_9176*, *gene_9626*, *gene_10284*, *gene_10460*, *gene_10489*, *gene_11449*,

*gene_12013*, *gene_12995*, *gene_14866*, *gene_15447*, *gene_15633*, *gene_15894*, *gene_15945*,

*gene_16817*, *gene_17688*, *gene_19236*

## Appendix B. List of 74 Selected Genes for the Adjacent-Categories Npo Model (See Section 4.3 and Section 4.5)

*gene_706*, *gene_742*, *gene_1510*, *gene_2288*, *gene_3439*, *gene_3461*, *gene_3552*,

*gene_3598*, *gene_3737*, *gene_3836*, *gene_3862*, *gene_4223*, *gene_4467*, *gene_4618*,

*gene_4640*, *gene_4833*, *gene_4979*, *gene_5050*, *gene_5394*, *gene_6162*, *gene_6226*,

*gene_6722*, *gene_6838*, *gene_6890*, *gene_7235*, *gene_7560*, *gene_7792*, *gene_7964*,

*gene_7965*, *gene_8003*, *gene_8349*, *gene_8891*, *gene_9175*, *gene_9176*, *gene_9181*,

*gene_9626*, *gene_9680*, *gene_9979*, *gene_10061*, *gene_10284*, *gene_10460*, *gene_10489*,

*gene_10809*, *gene_10950*, *gene_11440*, *gene_11449*, *gene_11566*, *gene_12013*, *gene_12068*,

*gene_12695*, *gene_12977*, *gene_12995*, *gene_13210*, *gene_13497*, *gene_14569*, *gene_14646*,

*gene_14866*, *gene_15447*, *gene_15633*, *gene_15894*, *gene_15896*, *gene_15898*, *gene_15945*,

*gene_16169*, *gene_16246*, *gene_16337*, *gene_16392*, *gene_16817*, *gene_17688*, *gene_17801*,

*gene_17949*, *gene_19236*, *gene_19661*, *gene_20476*

## Appendix C. Fitted Adjacent-Categories Npo Model with 74 Selected Genes (See Section 4.4)

As a special case of Model (3), the fitted (main-effects) adjacent-categories npo model with 74 selected genes in Section 4.4 can be written as

$$\log\left(\frac{{\hat{\pi }}_{ij}}{{\hat{\pi }}_{i,j+1}}\right)={\hat{\beta }}_{j1}+\sum_{k=2}^{75}{\hat{\beta }}_{jk}x_{i,k-1}$$

where i=1,…,801 is the sample index; j=1 (BRCA), 2 (COAD), 3 (KIRC), and 4 (LUAD) (thus ${\hat{\pi }}_{i5}$ represents PRAD); ${\hat{\beta }}_{jk}$ variables are estimated parameters listed in Table A1, Table A2 and Table A3; and $x_{i,k-1}$ is the expression level of the (k−1)th selected gene listed in Appendix B.

**Table A1:** Fitted parameters for the adjacent-categories npo model with 74 selected genes (Part I).

*k*	Name	j=1	j=2	j=3	j=4
1	*Intercept*	−19.842	−1.624	9.602	−10.581
2	*gene_706*	−0.232	0.0232	−0.592	0.281
3	*gene_742*	−0.549	−0.651	−0.406	0.355
4	*gene_1510*	0.0648	0.0107	0.0758	−0.344
5	*gene_2288*	−0.791	−0.277	−0.493	1.134
6	*gene_3439*	0.113	0.897	−0.997	−0.00296
7	*gene_3461*	0.272	0.028	−0.389	0.531
8	*gene_3552*	0.235	−0.398	0.428	−0.432
9	*gene_3598*	0.225	−0.142	−0.583	0.983
10	*gene_3737*	0.431	−0.394	0.0109	0.827
11	*gene_3836*	0.297	−0.546	0.538	−0.162
12	*gene_3862*	−0.463	0.207	−0.372	0.0824
13	*gene_4223*	−0.521	0.313	−1.592	1.269
14	*gene_4467*	0.167	0.139	0.500	−0.673
15	*gene_4618*	−0.669	0.358	−2.068	1.973
16	*gene_4640*	−0.738	0.711	−0.173	0.168
17	*gene_4833*	0.286	0.317	−0.611	0.229
18	*gene_4979*	−0.121	0.390	−1.267	1.178
19	*gene_5050*	−0.100	0.201	−0.256	0.222
20	*gene_5394*	0.124	0.150	−1.162	1.046
21	*gene_6162*	−1.111	−0.530	−0.306	0.327
22	*gene_6226*	0.135	0.130	0.663	−0.826
23	*gene_6722*	−0.392	0.356	−0.234	0.0363
24	*gene_6838*	0.525	−0.276	0.597	−0.494
25	*gene_6890*	−0.159	0.0228	−0.112	−0.333

**Table A2:** Fitted parameters for the adjacent-categories npo model with 74 selected genes (Part II).

*k*	Name	j=1	j=2	j=3	j=4
26	*gene_7235*	−1.114	0.178	−0.0569	0.193
27	*gene_7560*	0.160	−0.275	0.206	−0.392
28	*gene_7792*	0.331	−0.0609	−0.253	0.430
29	*gene_7964*	1.010	0.529	−0.142	−0.498
30	*gene_7965*	1.559	0.139	0.745	−0.534
31	*gene_8003*	0.042	−0.319	0.188	−0.185
32	*gene_8349*	0.488	−0.235	−0.0766	0.273
33	*gene_8891*	0.371	0.111	1.253	−1.249
34	*gene_9175*	0.0943	−0.219	1.001	0.192
35	*gene_9176*	0.0486	0.211	−1.320	0.985
36	*gene_9181*	0.0786	−0.196	0.139	−0.159
37	*gene_9626*	0.429	0.0889	0.204	0.169
38	*gene_9680*	−0.189	0.00538	0.364	−0.354
39	*gene_9979*	−0.201	−0.139	0.904	−0.950
40	*gene_10061*	−0.544	0.389	−1.463	0.909
41	*gene_10284*	−0.0725	0.0631	−0.593	0.712
42	*gene_10460*	0.526	−1.255	−0.416	1.049
43	*gene_10489*	−0.862	0.466	−0.206	0.231
44	*gene_10809*	−0.0157	0.00227	−0.662	0.613
45	*gene_10950*	0.127	0.219	−0.186	0.244
46	*gene_11440*	−0.548	0.385	0.0711	−0.231
47	*gene_11449*	0.366	0.0614	−0.336	−0.542
48	*gene_11566*	0.0015	−0.958	0.320	0.210
49	*gene_12013*	0.810	−0.662	−0.391	0.499
50	*gene_12068*	0.233	0.171	0.477	−0.391

**Table A3:** Fitted parameters for the adjacent-categories npo model with 74 selected genes (Part III).

*k*	Name	j=1	j=2	j=3	j=4
51	*gene_12695*	0.544	−0.276	0.426	−0.101
52	*gene_12977*	−0.173	0.319	−1.094	0.702
53	*gene_12995*	−0.0335	−0.032	−0.0601	0.207
54	*gene_13210*	0.736	−0.422	1.404	−0.650
55	*gene_13497*	0.0561	−0.175	0.335	−0.142
56	*gene_14569*	0.374	−0.000751	0.077	−0.262
57	*gene_14646*	0.269	−0.328	−0.199	0.212
58	*gene_14866*	−0.00596	0.307	1.347	−1.000
59	*gene_15447*	−0.209	0.00293	0.0196	0.0194
60	*gene_15633*	−0.000597	−0.0341	−0.0535	−0.122
61	*gene_15894*	−0.0516	−0.309	0.732	−0.596
62	*gene_15896*	−0.0755	0.0453	0.268	−0.332
63	*gene_15898*	0.640	0.272	0.674	−0.676
64	*gene_15945*	−1.183	0.683	0.232	−0.576
65	*gene_16169*	−0.633	0.213	−0.944	0.848
66	*gene_16246*	−0.248	0.646	−1.134	0.682
67	*gene_16337*	−0.384	−0.0586	0.373	−0.220
68	*gene_16392*	0.529	0.680	0.118	−0.271
69	*gene_16817*	0.0321	−0.0286	−0.127	−0.353
70	*gene_17688*	1.044	−0.438	1.351	−1.215
71	*gene_17801*	0.680	−0.0994	0.475	−0.0449
72	*gene_17949*	0.357	0.176	0.356	−0.456
73	*gene_19236*	−0.117	−0.0792	−0.0906	−0.173
74	*gene_19661*	−0.280	0.325	−0.501	0.327
75	*gene_20476*	0.151	−0.0272	0.597	−0.542
